# Application and evaluation of teaching practical histology with the use of virtual microscopy

**DOI:** 10.1186/1746-1596-8-S1-S7

**Published:** 2013-09-30

**Authors:** Eva Pospíšilová, Drahomíra Černochová, Radka Lichnovská, Běla Erdösová, Dimitrolos Krajčí

**Affiliations:** 1 Department of Histology and Embryology, Faculty of Medicine and Dentistry, Palacký University, Hněvotínská 3, 77515 Olomouc, Czech Republic

## Background

Emerging digital microscopy technology and newly developed scanning light microscopy systems enable histologists to transfer analog image data of entire slides into digital ones, and to provide them to students in the class with identical contents and quality [1,2]. We have recently started a major innovation of our system of practical teaching using e-learning methods of delivery of high quality histology virtual slides and supporting documents [3]. We have created our own teaching environment in a computer-equipped and networked classroom dedicated to histology practical sessions. This method of teaching has been complemented with electronic in-course and final assessments of student's theoretical and practical knowledge and skills.

## Material and methods

Glass histology slides have been scanned with Olympus dotSlide scanning system creating thousands of overlapping images saved in a proprietary file format. The scanning was done with standard 40x objective lens. Using dedicated viewer software (Olyvia, Olympus) the images were viewed on student’s PC as a single image map at variable magnifications. Our classroom, dedicated to histology practical sessions, consists of 1 server PC (teacher) and 30 client’s PC (students) [3]. The server PC has four accounts set up. Administrator account - with full administrator’s rights, Lector account - with partially limited power user’s rights. Student account and Exam account – with limited user’s rights, enable remote access of student’s workstations to the server. Under lector’s login, the supporting documents and virtual slides can be exchanged, edited, added or deleted for incremental updates.

The student’s PCs are standard ultraslim units running on Windows XP, SP3 operating system, under strict administrator’s control with Microsoft Steady State software. Client computers have also Administrator, Lector, Student, and Exam accounts set up. Students can access only the last two accounts without any login password. When opening the Student account, they are presented with a welcome screen where they can select their preferable language of instruction for practical class (Czech or English).

The core of this virtual slide learning system is our own Database of Histology Practical saved on the X:\drive of the server PC. It is build up in MS Excel format having the first contents page, followed by practical pages devoted to all 24 topics of histology practical [3]. For electronic testing of student’s practical knowledge we used special software Quizmaker ’09 (Articulate) which is simple and easy to use by teachers having no programming skills. Since this software has a selective option to shuffle sequences of questions and also to shuffle all distracters in the quiz randomly on monitors of examined students, only one version of histology test was enough to prepare for one practical class. A time limit for display of each question and a total time allowed for a complete test were also settable.

## Results and discussion

### Evaluation of practical sessions with virtual slides

Students of histology practical classes readily accepted the use of computers for observation of virtual slides. Using our own specific set of evaluation questions, we have asked students to evaluate this new method of practical sessions. Majority of students in General Medicine and Dentistry specializations (93%) evaluated positively the use of virtual slides, as they allowed them to study and also to discuss various details of cells and tissues clearly at various magnifications. About half of students (58%) claimed that they benefited from using the attached supporting documents during practical sessions and almost all students (96%) downloaded these supporting documents to their external media for later self-revisions. The classical light microscopes and glass slides, that were available next to computers, were used by histology students occasionally, according to their reply.

Teachers benefited from a uniform quality of presented slides and also from a straightforward and easy personal communication with students in the class when personal guidance and explanation was needed at student’s monitors. This was a highly beneficial feature when this system was used in large practical labs of 50 and more students participating simultaneously. PC-based classes of practical histology also provided an easy environment for computerized testing of student’s practical knowledge of structures displayed on their monitors.

### Virtual slides versus classical light microscopy

With the advent of virtual microscopy, the format of histology practical labs is changing towards the use of virtual slides viewed by individual students on their PCs. In the earlier-equipped histology practical labs the availability of both classical light microscopes and virtual slides is maintained up to date in order to give students a possibility to observe histology slides in both ways. According to survey of Drake et al. 2009 [4] regarding the laboratory experience of the 45 respondents, 13 reported that their laboratory used classical microscopes, 20 reported that their laboratory used virtual microscopy only, and 12 reported that their laboratory used a combination of microscopes and virtual microscopy. The authors conclude that histology lends itself to approaches that are more independent study friendly. This is especially true with the continuously increasing usage of virtual microscopy systems that students can access anywhere by computer.

## Conclusions

The e-learning format of histology practical based on virtual slides is a didactically efficient method of teaching histology. It standardizes the set of histology slides and gives all students in a large teaching group equal opportunity to see the same high quality slides in practical sessions. It also gives students a new experience with the observation of histology structures on PC monitors, and enables them to take selected screen prints of virtual slides for their own study/research observations as if they would be using a complete research microscope with a digital camera. Students evaluate positively their use of virtual slides and accompanying supporting documents in histology practical sessions. Teachers benefit from a uniform quality of presented slides and also from a straightforward and easy didactic communication with students in the class.

## List of abbreviations

PC: personal computer; MCQ: multiple choice question

## Competing interests

The authors declare that they have no competing interests in relation to this work.

## Authors' contributions

EP, DČ and RL participated in selection of glass slides for scanning, writing Czech versions of supporting documents, editing data in the Histology Practical Database, application and evaluation of virtual slides in practical sessions, and preparing MCQs for examinations. BE participated in editing English versions of supporting documents, application and evaluation of virtual slides in practical sessions, and preparing English versions of MCQs for examinations. DK conceived this project, planned and coordinated all works, scanned virtual slides, designed the Histology Practical Database, wrote English versions of supporting documents and trained the collaborating staff in use of the special software. All authors read and approved the final manuscript.
